# Supplementary material for: Binding of Immunoglobulin G to Protoporphyrin IX and Its Derivatives: Evidence the Fab Domain Recognizes the Protoporphyrin Ring
Source: Antibodies (Basel). 2019 Jan 4;8(1):6. doi: 10.3390/antib8010006 (PMC6640705; doi:10.3390/antib8010006)
Supplement: Supplementary file 1 [file antibodies-08-00006-s001.pdf]

antibodies

Supplementary: Binding of Immunoglobulin G to Protoporphyrin IX and Its Derivatives: Evidence the Fab Domain Recognizes the Protoporphyrin Ring

Koichi Orino

Laboratory of Veterinary Biochemistry, School of Veterinary Medicine, Kitasato University, Aomori 034-8628, Japan; orino@vmas.kitasato-u.ac.jp; Tel: +81-176-23-3471; Fax: +81-176-23-8703

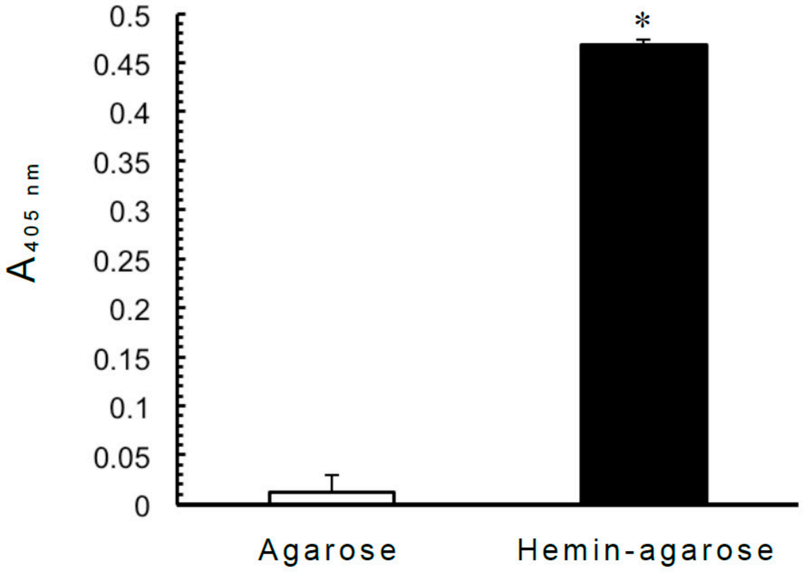

Figure 1. Binding of ALP-labeled anti-bovine Tf antibodies to hemin-agarose beads. Hemin-agarose and agarose were purchased from Sigma Chemical Co. (St. Louis, MO, USA). A 1-mL volume of PBS containing ALP-conjugated anti-Tf antibodies (25 μg), prepared according to a previously described method (Orino et al. 1993; Watanabe et al. 1994), and hemin-agarose beads or agarose beads in PBS (net volume of beads per sample: 25 μL each) was added to a Spitz glass tube masked with 0.1% (*w*/*v*) BA. The mixture was centrifuged at 1700× *g* for 15 min, and the pelleted beads were then washed three times with 1 mL of PBS. After washing, the pelleted beads were re-suspended with 1 mL of 3 mM disodium *p*-nitrophenyl phosphate. After incubation at 37 °C, the mixture was centrifuged at 1700× *g* for 5 min at room temperature, and the absorbance of the resulting supernatant was measured at 405 nm. Each value is the mean ± SD of three tests. * $p < 0.01$ versus binding examined with agarose beads.

© 2019 by the author. Licensee MDPI, Basel, Switzerland. This article is an open access article distributed under the terms and conditions of the Creative Commons Attribution (CC BY) license (http://creativecommons.org/licenses/by/4.0/).
